# Effect of armed conflict on measles surveillance and zero-dose vaccination status in Tigray, Ethiopia, 2018–2024

**DOI:** 10.1186/s13031-026-00827-9

**Published:** 2026-08-03

**Authors:** Habtamu Milkias Wolde, Ali Raza, Youngoh Bae, Seung Won Lee

**Affiliations:** 1 Department of Precision Medicine, School of Medicine, Sungkyunkwan University, Suwon, 16419 Republic of Korea; 2 Department of Neurosurgery, Korean Armed Forces Capital Hospital, Seongnam, 13487 Republic of Korea; 3 Department of Metabiohealth, Sungkyunkwan University, Suwon, 16419 Republic of Korea; 4 Personalized Cancer Immunotherapy Research Center, Sungkyunkwan University School of Medicine, Suwon, 16419 Republic of Korea; 5 Department of Family Medicine, Kangbuk Samsung Hospital, Sungkyunkwan University School of Medicine, Seoul, 03181 Republic of Korea

**Keywords:** Measles, Armed conflict, Disease surveillance, Routine immunization, Vaccination coverage, Tigray, Ethiopia

## Abstract

**Background:**

Armed conflict disrupts health systems, undermining routine immunization and disease surveillance. These disruptions can delay outbreak detection and allow population susceptibility to accumulate unnoticed. This study assessed the impact of the 2020–2022 conflict in Tigray, Ethiopia, on measles epidemiology, focusing on surveillance reporting, age distribution of cases, and vaccination status.

**Methods:**

We conducted a retrospective longitudinal analysis of national case-based measles surveillance data from Ethiopia (2018–2024; *n* = 69,866). Changes in measles surveillance, age distribution, and zero-dose vaccination status across the pre-conflict (2018–2019), conflict peak (2020–2022), and post-conflict recovery (2023–2024) periods were examined. Two-way analysis of variance examined regional differences in age at infection across phases. Multivariable logistic regression estimated adjusted odds ratios (aORs) for confirmed measles cases being unvaccinated (zero-dose), using the pre-conflict period as the reference and adjusting for age and sex. Surveillance quality was assessed using demographic data completeness.

**Results:**

During the conflict peak, reported measles cases from Tigray declined to 0.01% of nationally reported cases, consistent with near-total surveillance collapse. After hostilities ended, a marked pediatric shift emerged, with the median age of infection in Tigray declining from 24.0 years during the conflict to 5.0 years in the post-conflict period (*p* < 0.0001), a pattern not observed in other regions. Compared with the pre-conflict baseline, the odds that a confirmed measles case was zero-dose were substantially higher during the conflict peak (aOR: 71.43; 95% CI: 14.1–1000.0) and remained elevated during post-conflict recovery (aOR: 2.49; 95% CI: 1.17–5.52). During the conflict peak, 50% of confirmed cases in Tigray lacked sex-disaggregated data.

**Conclusion:**

The conflict was associated with a near-complete collapse of measles surveillance, marked changes in the age distribution of reported cases, and an increased proportion of zero-dose measles cases. These findings highlight the need for resilient surveillance systems and targeted catch-up vaccination during post-conflict recovery.

**Supplementary Information:**

The online version contains supplementary material available at https://doi.org/10.1186/s13031-026-00827-9.

## Introduction

Armed conflict remains one of the most persistent challenges to population health, not only through direct violence but through its indirect effects on health systems and essential services. While armed violence causes immediate morbidity and mortality, the longer-term burden is often driven by the disruption of healthcare delivery, supply chains, and public health infrastructure, resulting in excess preventable deaths well beyond the period of active hostilities [1, 2]. Conflict-related displacement, overcrowding, and destruction of facilities further exacerbate vulnerability to infectious diseases, particularly among children [3, 4]. Routine childhood immunization is especially susceptible to disruption, as it depends on stable logistics, functioning cold-chain systems, and consistent access to health workers - conditions that are frequently compromised during conflict [4, 5].

Measles is among the most sensitive indicators of such systemic breakdowns. Due to its high transmissibility, sustained interruption of transmission requires very high population immunity, typically exceeding 95% vaccination coverage [6, 7]. Even short-lived declines in immunization coverage can therefore result in outbreaks, making measles a reliable marker of gaps in routine service delivery [1, 3]. In conflict-affected settings, disruptions to cold-chain infrastructure caused by power shortages or fuel constraints may render available vaccines ineffective, while displacement into crowded and resource-limited environments facilitates rapid transmission among undernourished and unvaccinated children [4, 5]. As a result, measles outbreaks often reflect broader failures in health system functioning rather than isolated programmatic weaknesses.

Beyond immunization, armed conflict substantially weakens disease surveillance and outbreak response capacity. Effective surveillance depends on continuous reporting from peripheral health facilities to central authorities, supported by functional communication networks and laboratory systems [8–10]. In conflict zones, health facilities may be destroyed or abandoned, health workers displaced, and telecommunications disrupted, leading to prolonged interruptions in case reporting [10, 11]. This produces periods of apparent epidemiological under-reporting and lack of surveillance visibility, during which reported incidence declines despite ongoing transmission [8, 12]. Such surveillance gaps hinder timely response and obscure the true scale and distribution of disease, allowing susceptibility to accumulate undetected until services are partially restored [9, 13].

Ethiopia has historically made substantial investments in strengthening its primary healthcare system, including expansion of the health extension program and improvements in routine immunization coverage [10, 12]. Prior to 2020, the Tigray region was considered one of the better-performing regions in terms of health service delivery and surveillance capacity [11, 12]. The outbreak of armed conflict in late 2020, however, precipitated a severe and localized collapse of health services. The conflict was characterized by widespread destruction of facilities, interruption of medical supply chains, and prolonged blackouts of electricity and telecommunications, effectively isolating the region from national health systems [12, 14].

Following the cessation of large-scale hostilities in late 2022, attention has increasingly turned to the longer-term public health consequences of the conflict. While national disease control programs continued to operate in much of Ethiopia, Tigray experienced a prolonged period of limited-service restoration. As surveillance systems gradually resumed, emerging data suggested substantial changes in measles epidemiology that could not be readily explained by national trends alone. However, the extent to which these patterns reflect true shifts in disease burden versus delayed detection of previously hidden susceptibility remains poorly understood.

To address this gap, we analyzed seven years (2018–2024) of national measles case-based surveillance data to examine changes in measles epidemiology in Tigray before, during, and after the conflict. By comparing age distribution, surveillance completeness, and vaccination status in Tigray with the rest of Ethiopia, this study aims to quantify how conflict-related disruption of immunization and surveillance systems altered the observable epidemiology of measles and left a cohort of children vulnerable to post-conflict outbreaks.

## Methodology

### Study setting and period

**Fig. 1:**
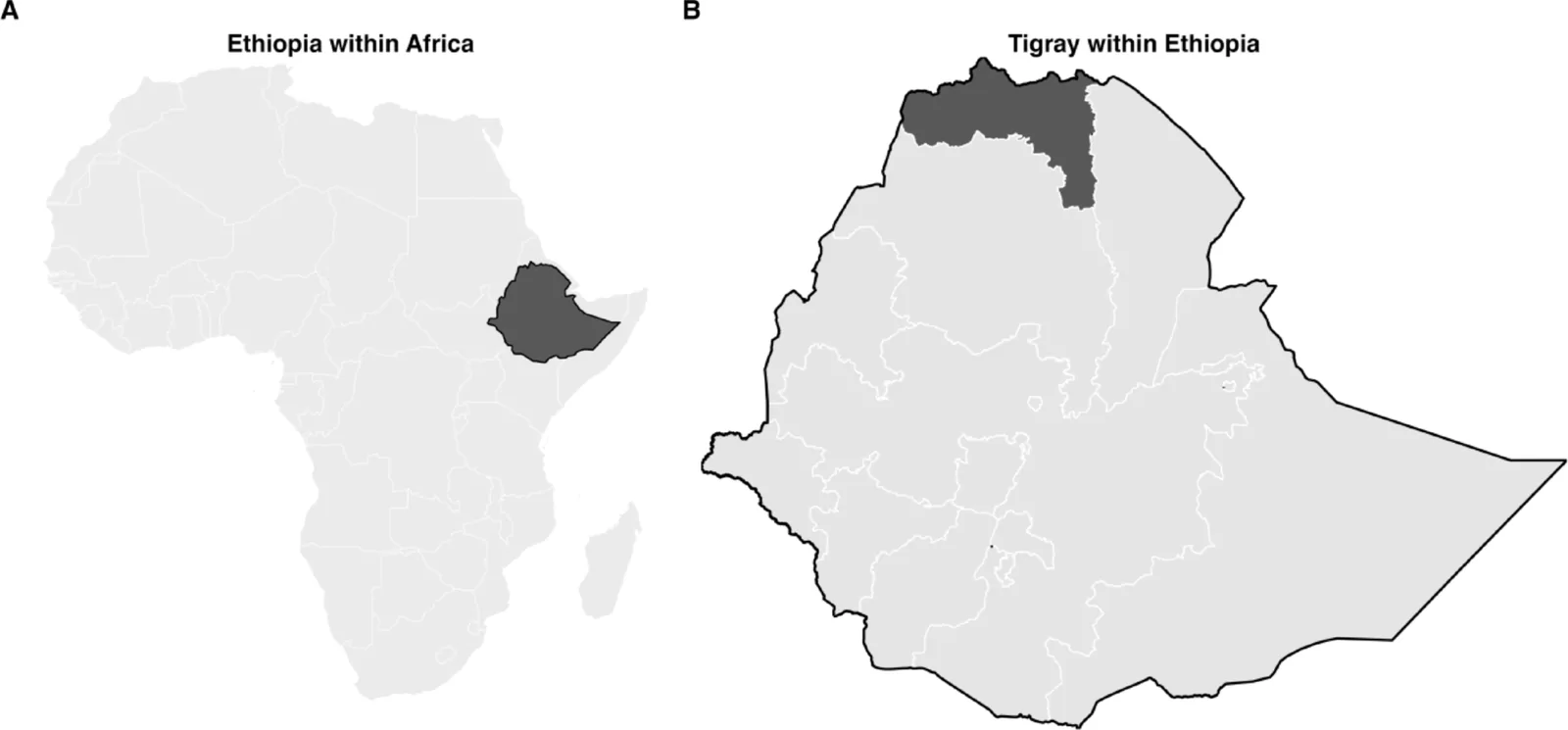
Study setting. (**A**) Location of Ethiopia within Africa. (**B**) Location of the Tigray Regional State within Ethiopia. The map is provided to illustrate the geographic setting of the study. Source: Authors’ illustration using Natural Earth country boundary data (https://www.naturalearthdata.com) and the Ethiopia administrative boundary dataset from Global Administrative Areas (GADM), version 4.1 (https://gadm.org)

Tigray is the northernmost regional state of Ethiopia. This study analyzed national case-based measles surveillance data collected between 2018 and 2024, encompassing the pre-conflict period (2018–2019), the armed conflict (2020–2022), and the post-conflict recovery period (2023–2024). Armed conflict began in November 2020 and continued until the signing of the Cessation of Hostilities Agreement in November 2022. During this period, widespread disruption of healthcare delivery, population displacement, and interruptions to routine public health services were reported across much of the region (Fig. 1).

### Study design and data source

We conducted a retrospective, population-based cohort analysis utilizing the national measles case-based surveillance database of Ethiopia. This dataset is maintained by the Ethiopian Public Health Institute (EPHI) and serves as the primary repository for the Integrated Disease Surveillance and Response (IDSR) system. Surveillance data capture reported cases and do not estimate true incidence. The study period, spanning from January 2018 to December 2024 in Gregorian calendar, was selected to capture a robust pre-conflict baseline, the active conflict phase, and the subsequent recovery period.

### Case classification and inclusion criteria

Cases were systematically included based on the World Health Organization (WHO) and EPHI standardized case definitions [15, 16]. Laboratory-confirmed cases were defined by clinical symptoms of measles (fever, maculopapular rash, and at least one of cough, coryza, or conjunctivitis) accompanied by a positive measles-specific IgM antibody test. Epidemiologically linked cases were those meeting the clinical definition with a direct temporal and geographical link, within 30 days, to a laboratory-confirmed case. To maintain the highest specificity for our demographic analysis and mitigate misclassification with other febrile rash illnesses during conflict-driven surveillance disruptions, we excluded cases classified as “Discarded” (IgM negative) or those identified solely as “Clinically Compatible” without laboratory or epidemiological linkage.

### Study variables and conflict period definition

The primary independent variable was conflict period, categorized a priori into three epidemiologically relevant phases based on the timing of the armed conflict and subsequent recovery: pre-conflict (2018–2019), conflict peak (2020–2022), and post-conflict recovery (2023–2024). The Pre-Conflict period (2018–2019) represents a baseline of stable routine immunization and active surveillance. The Conflict Peak (2020–2022) is characterized by the onset of hostilities, widespread disruption of cold-chain infrastructure, and the systemic collapse of health reporting in affected areas [17, 18]. Finally, the Post-Conflict/Recovery phase (2023–2024) marks the cessation of hostilities and the re-establishment of the national reporting system alongside supplemental immunization activities (SIAs). All dates and study periods were defined using the Gregorian calendar, and annual case counts were aggregated from January through December of each calendar year. Geographic location (region) was also included as an independent variable for comparative analyses.

The study evaluated three primary outcomes. First, surveillance performance was assessed using the annual number and proportion of laboratory-confirmed and epidemiologically linked measles cases reported from Tigray relative to nationally reported cases. Second, age at infection was analyzed as a continuous variable (years) to examine demographic changes in reported measles cases across conflict periods. Third, vaccination status was derived from routine case-based measles surveillance records, which document the number of measles-containing vaccine (MCV) doses received before illness onset. Confirmed cases with no documented measles vaccine dose were classified as zero-dose, whereas those with one or more documented doses were classified as vaccinated. Cases with missing vaccination information were categorized separately as unknown and excluded from regression analyses. For this study, zero-dose was operationally defined as confirmed measles cases with no documented receipt of an MCV prior to infection and it differs from Gavi’s programmatic definition of zero-dose children, which is based on the absence of DTP1 vaccination in the first year of life.

For multivariable analyses, conflict period was the principal exposure variable. Age (continuous) and sex were included as covariates because of their established associations with measles epidemiology and vaccination status.

Variables were selected a priori based on epidemiological relevance and availability within the case-based surveillance dataset. Conflict phase was the principal exposure, while age and sex were included as potential confounders because both may be associated with documented measles vaccination status. The model was restricted to confirmed cases from Tigray; therefore, region was not included as a covariate. Other potentially relevant variables, including displacement status, socioeconomic characteristics, nutritional status, access to health services, and district-level conflict intensity, were either not collected or were too incomplete for reliable inclusion. A parsimonious model was therefore used to reduce the risk of unstable estimates and overfitting, particularly given the small number of reported cases during the conflict period.

### Spatial harmonization and administrative rescaling

During the study period, Ethiopia underwent significant administrative restructuring, notably the dissolution of the Southern Nations, Nationalities, and Peoples’ Region (SNNPR) into several smaller regional states. To ensure longitudinal consistency, we performed manual spatial harmonization by redistributing cases originally coded under SNNPR into the new regional entities - South Ethiopia, Central Ethiopia, South West Ethiopia, and Sidama - by cross-referencing zonal and woreda-level geographic identifiers with the 2023 administrative decree. This spatial harmonization was critical to prevent geographical artifacts from confounding regional comparisons and ensuring that national benchmarks remained accurate over time.

### Demographic disaggregation and vaccination status classification

To characterize demographic shifts within the affected population, we summarized patient characteristics using descriptive statistics stratified by vaccination status. Confirmed measles cases with no documented receipt of a measles-containing vaccine before infection were classified as Zero-Dose, whereas those with documentation of one or more doses were classified as ≥ 1 Dose. The latter category represents prior contact with measles immunization services but does not necessarily indicate vaccine failure, as it may also reflect delayed vaccination, incomplete documentation, cold-chain disruption, waning immunity, or other operational challenges affecting immunization delivery [11]. These cohorts were disaggregated by biological sex-standardized to title-case to address reporting “sparsity” and three age brackets (infants < 9 months, target pediatric 9–59 months, and older cohorts 5 + years), with continuous age analysis utilizing the median and interquartile range (IQR) to account for the non-normal, multi-modal distribution observed in the raw data. Notably, missing demographic data, which exceeded 50% for biological sex in Tigray during the conflict peak, was treated as a sentinel indicator of surveillance reporting strain rather than simple random omission, and was addressed through a combination of complete-case analysis for disaggregation and stratified median imputation for age to mitigate sentinel bias.

### Addressing informative missingness and sentinel bias

Surveillance data in conflict zones frequently suffer from informative missingness, where the probability of data being missing is directly related to conflict intensity. During the conflict peak, surveillance in Tigray was largely restricted to mobile adults or individuals able to access the few functioning urban health facilities, resulting in sentinel bias. To address this, we employed a stratified median imputation strategy. Missing age values were replaced using the median age calculated within each region and conflict phase, a conservative approach chosen to preserve distributional robustness in the presence of right-skewed age distributions and to minimize distortion from adult-dominated reporting during the conflict period. This approach reduced the likelihood that conflict-related reporting patterns would bias age distributions observed during the recovery phase.

All ages were converted to decimal years, incorporating months for infants, to improve precision in the 0–5-year age group, where the epidemiological impact of missed routine immunization is most pronounced.

### Statistical analysis and interaction modeling

To mathematically evaluate the shift in transmission, we employed a Two-Way Analysis of Variance (ANOVA) framework within a linear interaction model (Age ~ Region x Phase). This model assessed the significance of the interaction between the region and the conflict phase to determine if Tigray’s demographic shift was a localized anomaly compared to the national baseline. Non-parametric analysis was further supported by the Wilcoxon Rank Sum test to compare median ages between the Conflict Peak and Post-Conflict periods [19].

To quantify the odds that a confirmed measles case was zero-dose, we developed a multivariable logistic regression model to calculate adjusted Odds Ratios (aOR) and 95% Confidence Intervals (CI) for Zero-Dose (unvaccinated) status. The model was adjusted for the primary exposure (Conflict Phase) and potential confounders, including age and sex. By setting the Pre-Conflict phase as the reference group, we isolated the relative increase in the likelihood of a confirmed case being unvaccinated during the post-conflict resurgence. These estimates are conditional on reported cases and do not represent population-level vaccination coverage. Multicollinearity among explanatory variables was assessed using variance inflation factors (VIFs). All variables had VIF values below 2, indicating no evidence of problematic multicollinearity (Supplementary Table S5**)**. Finally, to evaluate the robustness of the primary findings, we performed a sensitivity analysis using an alternative temporal classification in which the study period was categorized as pre-conflict (2018–2020), conflict period (2021–2022), and post-conflict recovery (2023–2024). The principal analyses, including descriptive statistics, two-way analysis of variance, multivariable logistic regression, and surveillance reporting summaries, were repeated using this alternative classification. Detailed results are presented in the Supplementary Material. All analyses were conducted in R (Version 4.5.1) using the tidyverse, lubridate, and broom packages [20]. This study is reported in accordance with the STROBE reporting guidelines for observational studies.

## Results

### The reporting void and surveillance blackout

The analysis reveals a profound divergence between national epidemiological trends and regional reporting in Tigray during the conflict period. Between 2018 and 2020, Tigray maintained a stable reporting baseline, contributing up to 4.5% of the national measles case-load. However, following the onset of hostilities, a “statistical blackout” occurred: while national cases surged from 1,884 in 2021 to 8,482 in 2022, Tigray’s reported cases plummeted to a low of just 1 case in 2022 (Table 1).

**Fig. 2:**
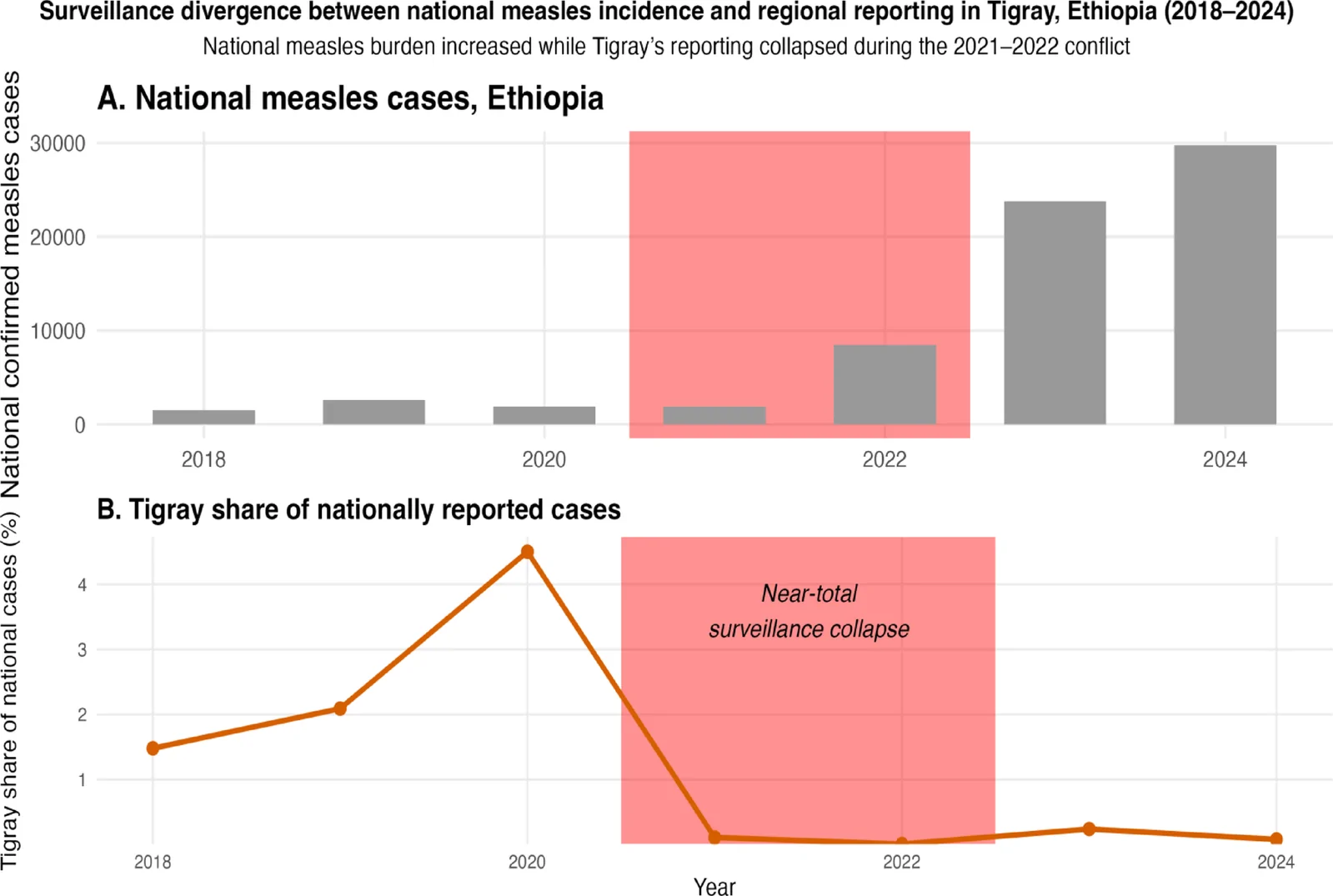
Divergence between the national measles epidemic and surveillance reporting from Tigray, Ethiopia, 2018–2024.** (A)** Annual number of laboratory-confirmed and epidemiologically linked measles cases reported nationally, demonstrating the substantial increase in measles incidence during 2021–2024. **(B)** Tigray’s annual contribution to nationally reported confirmed measles cases, expressed as the percentage of all reported cases. The shaded area denotes the 2021–2022 conflict period, during which Tigray’s contribution declined to nearly zero despite increasing national measles transmission, indicating a near-total collapse of surveillance reporting

This represents a drop in regional reporting efficiency to 0.01%, signaling a near-total collapse of the IDSR system during the conflict peak. Despite a nationwide epidemic expansion in 2023 and 2024 (reaching nearly 30,000 cases), Tigray’s reporting share remained suppressed at < 0.1%, indicating a lingering surveillance gap during the recovery phase. Importantly, these reductions in reported cases should be interpreted as surveillance failure rather than evidence of reduced measles transmission during the conflict period. (Fig. 2).

**Table 1:** Characteristics of measles cases by region and conflict period

Year	National cases	Tigray cases *n* (%)	Status
2018	1,489	22 (1.48)	Baseline
2019	2,628	55 (2.09)	Baseline
2020	1,865	84 (4.5)	Conflict Onset
2021	1,884	2 (0.11)	System Collapse
2022	8,482	1 (0.01)	System Collapse
2023	23,762	57 (0.24)	Resurgence/Recovery
2024	29,756	24 (0.10)	Resurgence/Recovery

### The demographic shift in age distribution

Statistical analysis of the age distribution confirms a substantial demographic shift in the outbreak profile across the three defined phases. During the peak of the conflict (2020–2022), the median age of laboratory-confirmed measles cases in Tigray rose to 24.0 years, with a wide interquartile range (IQR: 16.0–32.0 years) (Table 2). This unusually adult-centered and diffuse distribution is unlikely to reflect a true change in measles epidemiology, but rather a profound distortion in case ascertainment, whereby the surveillance system predominantly captured mobile, symptomatic adults or cases presenting to the few functioning urban health facilities. In contrast, pediatric transmission - historically the core of measles epidemiology - was largely absent from official records during this period.

**Table 2:** Age structure of measles cases (Medians and IQR) by conflict phases

Comparison group	Conflict phases	Number of cases	Median age (Yrs.) [IQR]
Rest of Ethiopia	Pre-Conflict (2018-19)	4,040	7 [3–19]
	Conflict Peak (2020-22)	12,144	5 [2–13]
	Post-Conflict (2023-24)	53,437	4 [2–9]
Tigray	Pre-Conflict (2018-19)	77	19 [9–26]
	Conflict Peak (2020-22)	87	24 [6.5–31]
	Post-Conflict (2023-24)	81	5 [2–22]

Following the partial and gradual restoration of health services after the cessation of hostilities (2023–2024), the median age of infection dropped sharply to 5.0 years, and the age distribution became highly compressed within early childhood (IQR: 2.0–9.0 years), revealing a classic pediatric outbreak pattern. This abrupt reversion is unlikely to reflect a true epidemiologic transition. Instead, it indicates delayed detection of accumulated susceptibility. The contrast between the conflict and post-conflict age distributions was highly significant (Wilcoxon rank-sum test, *p* < 0.0001), reinforcing the magnitude of this demographic reversal.

**Fig. 3:**
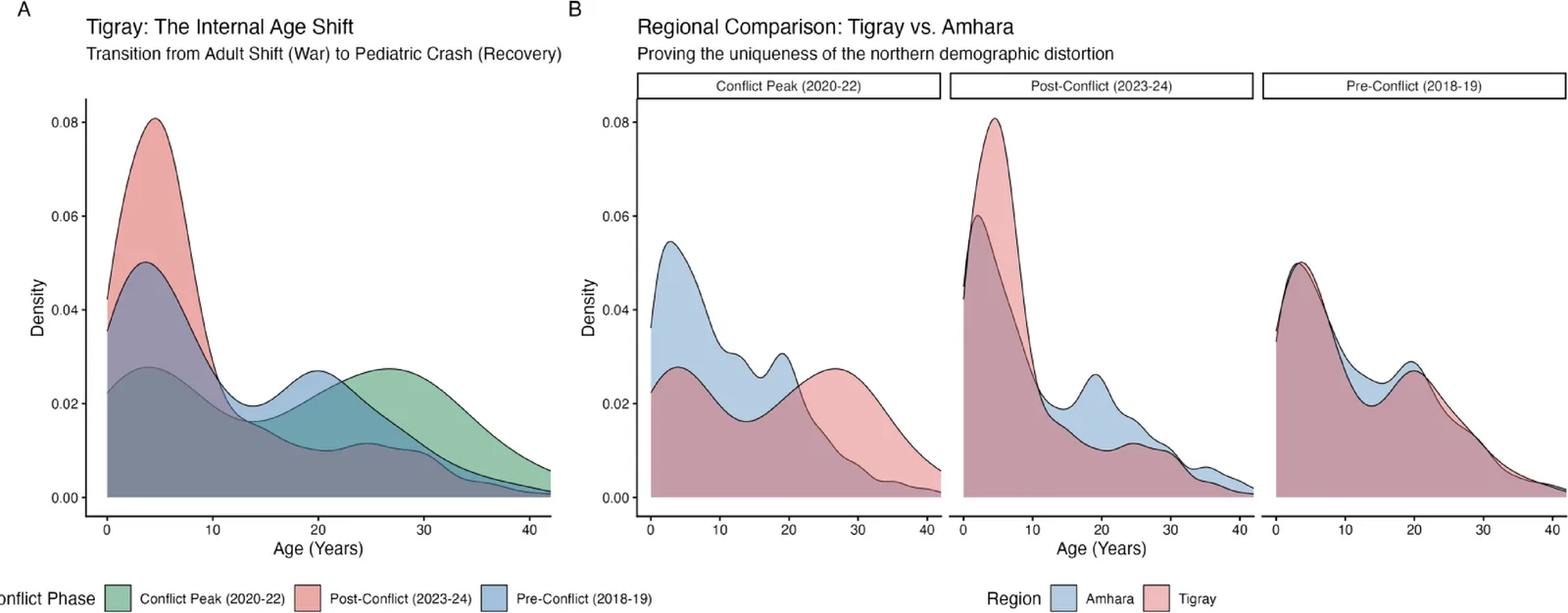
Age distribution of confirmed measles cases during conflict and post-conflict recovery in Tigray, Ethiopia. **(A)** Kernel density plots showing the age distribution of laboratory-confirmed measles cases in Tigray from 2018 to 2024. The distribution shifts from a predominantly pediatric pattern before the conflict to a broader, adult-skewed profile during the conflict period (2020–2022), followed by a marked return to younger age groups in the post-conflict period (2023–2024). **(B)** Comparative age distributions between Tigray and the neighboring Amhara region across the same periods. While Amhara demonstrates a stable, pediatric-dominated age profile throughout, Tigray shows a pronounced temporal distortion during the conflict, consistent with disruption of routine immunization and health service access

Comparison with the neighboring Amhara region - where a consistently pediatric-focused age distribution was maintained throughout the study period - further highlights the uniqueness of the Tigray experience. The absence of a similar demographic distortion in Amhara supports the interpretation that the observed shift in Tigray was driven by conflict-related disruptions to immunization and surveillance rather than broader national or seasonal changes in measles transmission. Together, these findings illustrate how prolonged service collapse can mask pediatric outbreaks and allow susceptibility to accumulate, only to be revealed once minimal surveillance and healthcare access are restored (Fig. 3).

To determine if the demographic shifts observed in Tigray were merely a reflection of national trends or a localized consequence of the conflict, we performed a Two-Way Analysis of Variance (ANOVA) examining the interaction between Region and Conflict phases. The model demonstrated a highly significant interaction effect (*F* (2, 69,860) = 94.3, *p* < 0.0001).

The interaction term for Tigray during the post-conflict period (2023–2024) was strongly and independently associated with a reduction in age at infection (*β* = −8.22, *p* < 0.0001). This effect indicates that the pronounced decline in the age of reported cases in Tigray - returning to a predominantly pediatric profile - was a statistically distinct phenomenon. The magnitude and direction of this interaction support the interpretation that the adult-skewed age distribution observed during the conflict represented a localized distortion in case detection rather than a true shift in transmission dynamics. Importantly, this correction occurred only after partial restoration of health services and surveillance and was not observed in other regions, where a stable pediatric age profile was maintained throughout the study period (Table 3).

**Table 3:** ANOVA table showing the interaction between Region and Conflict phases

Source of Variation	Sum of Squares	Degrees of freedom	Mean Squares	F-value	Pr. (> F)
Region (Tigray vs. Rest)	22,056	1	22,056	306.8	< 2.2 × 10^− 16^ ***
Phase (Conflict Phases)	67,812	2	33,906	471.6	< 2.2 × 10^− 16^ ***
Region × Phase Interaction	13,564	2	6,782	94.3	< 2.2 × 10^− 16^ ***
Residuals	5,022,934	69,860	71.9		

*** *P* < 0.0001

### Comparative analysis of measles zero-dose status

The proportion of zero-dose cases defined in this study as confirmed measles cases with no documented receipt of a measles-containing vaccine before infection was low in Tigray during the pre-conflict period (8.3%) but increased markedly during the conflict period (48.1%). In contrast, the corresponding proportion in the Rest of Ethiopia remained relatively stable, changing from 42.4% before the conflict to 37.3% during the conflict (Table 4).

During the post-conflict period, the proportion of zero-dose cases in Tigray declined to 39.5% but remained substantially higher than the pre-conflict level. In the Rest of Ethiopia, the corresponding proportion increased modestly to 40.3%. These findings indicate that, despite the recovery of surveillance reporting after the conflict, a substantial proportion of confirmed measles cases in Tigray continued to have no documented measles vaccination (Table 4).

**Table 4:** Comparison of zero dose vaccination proportion between Tigray and the rest of Ethiopia by conflict phases

Comparison group	Conflict phase	Zero-dose, *n*/*N* (%)	95% CI
Rest of Ethiopia	Pre-conflict (2018–2019)	1,674/3,945 (42.4%)	40.9–44.0
Rest of Ethiopia	Conflict period (2020–2022)	4,520/12,132 (37.3%)	36.4–38.1
Rest of Ethiopia	Post-conflict (2023–2024)	21,477/53,265 (40.3%)	39.9–40.7
Tigray	Pre-conflict (2018–2019)	5/60 (8.3%)	3.6–18.1
Tigray	Conflict period (2020–2022)	38/79 (48.1%)	37.4–58.9
Tigray	Post-conflict (2023–2024)	32/81 (39.5%)	29.6–50.4

To isolate the impact of the conflict on immunization coverage while controlling for demographic confounders, we employed a multivariable logistic regression model with the Pre-Conflict period (2018–2019) as the reference baseline. The analysis revealed a profound temporal escalation in the risk of zero-dose status. During the conflict peak (2020–2022), the odds of a confirmed measles case being Zero-Dose were 71.4 times higher than the pre-war baseline (aOR: 71.43; 95% CI: 14.1–1000.0; *p* < 0.001), highlighting a near-total cessation of routine immunization services. Although recovery efforts were initiated in the post-conflict phase (2023–2024), the risk of being Zero-Dose remained significantly elevated at 2.5 times the pre-conflict level (aOR: 2.49; 95% CI: 1.17–5.52; *p* = 0.020).

Age functioned as a significant continuous predictor of vaccination status (aOR: 0.95; *p* < 0.001), where each additional year of age was associated with a 5% decrease in the likelihood of being Zero-Dose. This trend likely reflects the cumulative opportunities for vaccination afforded to older cohorts prior to the healthcare system’s collapse. Conversely, biological sex was not a significant predictor of immunization status (*p* = 0.474), with no statistically significant difference observed between male and female cases. This suggests that the susceptibility gap in Tigray was driven by systemic geographic and temporal barriers such as the regional blockade and cold-chain destruction rather than gender-based disparities in healthcare seeking (Table 5).

**Table 5:** Multivariable logistic regression analysis of factors associated with Zero-Dose (Unvaccinated) status among confirmed measles cases in Tigray region

Predictor variable	Adjusted odds ratio (aOR)	95% Confidence interval (CI)	*p*-value
Conflict phase			
Pre-Conflict (2018–19)	Reference	--	--
Conflict Peak (2020–22)	71.43	[14.1–1000.0]	< 0.001 ***
Post-Conflict (2023–24)	2.49	[1.17–5.52]	0.020 *
Age (Continuous)	0.95	[0.922–0.976]	< 0.001 ***
Sex			
Male	Reference	--	--
Female	0.78	[0.394–1.540]	0.474

162 (23.8%) female and 178 (26.2%) male confirmed measles cases from Tigray had missing sex values

### Sensitivity analysis

The sensitivity analysis using an alternative temporal classification produced findings that were consistent with the primary analyses. The marked decline in the age of confirmed measles cases following the conflict and the significant interaction between conflict period and geographic comparison group remained unchanged (Supplementary Tables S1 and S2). However, because only three confirmed measles cases were reported from Tigray during 2021–2022, the multivariable logistic regression model had insufficient statistical power to provide stable estimates of the association between conflict period and zero-dose vaccination status (Supplementary Table S3). The surveillance reporting analysis similarly confirmed that the most pronounced decline in reporting occurred during 2021–2022 (Supplementary Table S4).

## Discussion

This study examined the impact of the 2020–2022-armed conflict on measles surveillance and vaccination status in Tigray using national case-based surveillance data spanning the pre-conflict, conflict, and post-conflict periods. Three principal findings emerged. First, measles surveillance in Tigray nearly collapsed during the conflict, with the region contributing only 0.01% of nationally reported confirmed cases in 2022 despite a substantial national resurgence. Second, the age distribution of confirmed cases changed markedly following the conflict, with the median age declining from 24 years during the conflict period to 5 years during post-conflict recovery. Third, the proportion of confirmed measles cases with no documented measles vaccination increased substantially during the conflict and remained elevated during the recovery period. Together, these findings indicate that the conflict disrupted measles surveillance while substantial immunity gaps accumulated during a period of severely constrained health service delivery. The persistence of an older age distribution during the conflict, followed by an abrupt post-conflict shift toward younger children, suggests that a large susceptible pediatric population became apparent only after surveillance activities resumed. Likewise, the persistently elevated proportion of zero-dose confirmed cases during the recovery period indicates that susceptibility continued beyond the cessation of hostilities.

The demographic pattern observed in Tigray represents a distinct epidemiological signature of severe health system disruption. Although confirmed measles cases in Tigray already exhibited an older age distribution before the conflict, this adult-dominated profile persisted during the conflict despite widespread disruption of health services. Rather than reflecting a biological change in measles transmission, this pattern is more plausibly explained by a surveillance artifact resulting from the collapse of pediatric healthcare access and routine case detection, whereby severe cases presenting to the limited functioning referral facilities were disproportionately older children and adults [21, 22]. Under these conditions, the IDSR system primarily captured mobile, symptomatic adults who were able to reach the limited functioning urban health facilities, while pediatric cases in rural and inaccessible areas remained largely undetected. The subsequent decline in the median age of infection to five years following the cessation of hostilities reflects the delayed detection - or “unmasking” - of accumulated susceptibility among children who missed routine immunization during the conflict period [23, 24]. Similar patterns of concealed pediatric transmission have been documented in other conflict-affected settings, including Syria, where reporting efficiency temporarily decoupled from true disease burden during periods of insecurity [25].

The markedly increased odds of zero-dose status among confirmed cases in the post-conflict period further quantify the extent of this susceptibility gap. While these estimates are conditional on reported cases and should not be interpreted as population-level coverage measures, the magnitude of the observed disparity indicates a substantial interruption of routine immunization services. In other humanitarian crises, including Yemen and the Democratic Republic of the Congo, conflict-related disruptions have been associated with two- to five-fold increases in zero-dose prevalence [17, 26, 27]. The considerably larger effect observed in Tigray suggests a near-complete breakdown of vaccine delivery and likely cold-chain functionality during the conflict period, resulting in a prolonged accumulation of susceptible cohorts [4, 28]. In this context, measles case detection patterns reflect the spatial and temporal distribution of health service access rather than solely underlying transmission dynamics, consistent with prior evidence from settings experiencing prolonged blockades or service collapse [29].

The scale of the observed susceptibility gap indicates that restoration of routine immunization services alone is unlikely to be sufficient to prevent future outbreaks. Instead, targeted, age-stratified Supplemental Immunization Activities (SIAs) are required to address immunity gaps among children born during the conflict period, particularly those born between 2020 and 2023 who were systematically missed by routine services [30, 31]. Strengthening the resilience of the surveillance system is equally critical. Decentralized surveillance approaches, including community-based reporting and diagnostic capacity that does not rely exclusively on centralized laboratory infrastructure, may help reduce future reporting voids in fragile settings [21, 32]. Integration of vaccination and surveillance efforts with existing social protection mechanisms, such as the Productive Safety Net Program (PSNP), may further improve access to marginalized and hard-to-reach populations [31].

A key strength of this study is the use of a national comparative framework, allowing the epidemiological patterns observed in Tigray to be distinguished from broader national trends. By comparing Tigray with the rest of Ethiopia, we were able to isolate the conflict as the primary driver of the observed age redistribution, rather than attributing these changes to national shifts in measles epidemiology or seasonality [22]. Nevertheless, several limitations must be acknowledged. The absence of surveillance during 2021–2022 introduces substantial selection bias, with reported cases likely representing a small and atypical subset of the true burden, skewed toward mobile and urban populations. Although stratified median imputation was used to address missing age data, this approach cannot fully reconstruct epidemiological patterns lost during periods of complete reporting disruption [33]. In addition, the absence of reliable case-fatality data limits assessment of the mortality impact associated with the post-conflict pediatric resurgence.

## Conclusion

The 2020–2022 conflict in Tigray dismantled years of immunization gains, leading to a profound increase in zero-dose status and a conflict-specific shift in the age distribution of measles cases that revealed a large, previously undetected pediatric epidemic. Measles proved to be a sensitive indicator of health system breakdown: when surveillance collapsed, transmission persisted silently among vulnerable populations. Addressing this accumulated susceptibility will require urgent implementation of multi-cohort catch-up vaccination strategies and the development of decentralized, conflict-resilient surveillance systems to prevent future high-mortality outbreaks during post-conflict recovery.

## Supplementary Information

Supplementary Material 1

## Data Availability

The data used in this study are not publicly available due to national data protection regulations but can be obtained from the corresponding author upon reasonable request and with permission from the relevant national authorities.

## Abbreviations

ANOVA: Analysis of variance
EPHI: Ethiopian public health institute
CI: Confidence interval
IQR: Interquartile range
IDSR: Integrated disease surveillance and response
PSNP: Productive safety net program
SIA: Supplemental immunization activity
WHO: World health organization
