# Supplementary material for: Effect of armed conflict on measles surveillance and zero-dose vaccination status in Tigray, Ethiopia, 2018–2024
Source: Confl Health. 2026 Aug 3;20:87. doi: 10.1186/s13031-026-00827-9 (PMC13721506; doi:10.1186/s13031-026-00827-9)
Supplement: Supplementary file 1 [file 13031_2026_827_MOESM1_ESM.docx]

**Supplementary appendix**

**Sensitivity analysis**

To evaluate whether the primary findings were sensitive to the definition of the conflict period, we repeated the principal analyses using an alternative temporal classification suggested during peer review. In this sensitivity analysis, the study period was categorized as pre-conflict (2018 –2020), conflict period (2021–2022), and post-conflict recovery (2023–2024), thereby separating the first year of conflict from the period of greatest surveillance disruption. The same analytical approach used in the primary analysis was repeated, including descriptive analyses, two-way analysis of variance (ANOVA), multivariable logistic regression, and surveillance reporting summaries.

**Supplementary Table S1. Age distribution of confirmed measles cases in Tigray using an alternative temporal classification.**

| **Conflict period** | **n** | **Median age (years)** | **IQR** | **25th percentile** | **75th percentile** |
| --- | --- | --- | --- | --- | --- |
| Pre-conflict (2018–2020) | 161 | 21 | 22 | 6 | 28 |
| Conflict period (2021–2022) | 3 | 23 | 7 | 16 | 23 |
| Post-conflict (2023–2024) | 81 | 5 | 20 | 2 | 22 |

The alternative classification produced results consistent with the primary analysis. Median age remained elevated during the conflict period and declined markedly during the post-conflict period, supporting the robustness of the observed post-conflict pediatric shift.

**Supplementary Table S2. Two-way ANOVA evaluating age at infection using the alternative conflict-period classification.**

| **Source** | **Degrees of freedom** | **F** | **P-value** |
| --- | --- | --- | --- |
| Comparison group | 1 | 302.06 | <0.001 |
| Conflict period | 2 | 502.82 | <0.001 |
| Interaction | 2 | 10.01 | <0.001 |

The interaction between geographic comparison group and conflict period remained statistically significant under the alternative temporal classification, indicating that the age distribution in Tigray changed differently from the rest of Ethiopia regardless of the period definition.

**Supplementary Table S3. Sensitivity analysis of multivariable logistic regression using an alternative temporal classification.**

| **Variable** | **Adjusted OR** | **95% CI** | **P** |
| --- | --- | --- | --- |
| Conflict period (2021–2022) | 1.24 | 0.06–13.60 | 0.865 |
| Post-conflict (2023–2024) | 1.25 | 0.65–2.38 | 0.504 |
| Age (years) | 0.97 | 0.94–0.99 | 0.005 |
| Male sex | 0.87 | 0.47–1.60 | 0.656 |

Unlike the primary analysis, the alternative temporal classification produced only three confirmed cases during the 2021–2022 conflict period. Consequently, the regression model lacked sufficient statistical power to estimate the association between conflict period and zero-dose vaccination status reliably. The wide confidence intervals therefore reflect sparse data rather than evidence against an association.

**Supplementary Table S4. Annual contribution of Tigray to nationally reported confirmed measles cases under the alternative temporal classification.**

| **Year** | **National cases** | **Tigray cases** | **% National** |
| --- | --- | --- | --- |
| 2018 | 1489 | 22 | 1.48 |
| 2019 | 2628 | 55 | 2.09 |
| 2020 | 1865 | 84 | 4.50 |
| 2021 | 1884 | 2 | 0.11 |
| 2022 | 8482 | 1 | 0.01 |
| 2023 | 23762 | 57 | 0.24 |
| 2024 | 29756 | 24 | 0.08 |

The sensitivity analysis confirmed that surveillance reporting remained comparable to the pre-conflict period during 2020 but declined dramatically during 2021–2022, coinciding with the period of greatest disruption to surveillance activities.

**Supplementary Table S5. Assessment of multicollinearity for the sensitivity model.**

| **Variable** | **Adjusted GVIF** |
| --- | --- |
| Conflict period | 1.04 |
| Age | 1.05 |
| Sex | 1.02 |

All adjusted GVIF values were below 2, indicating no evidence of problematic multicollinearity.
